# Fecal microbiota transplantation is a promising therapy for kidney diseases

**DOI:** 10.3389/fmed.2025.1628722

**Published:** 2025-07-09

**Authors:** Jiawei Zhang, Xiangge Ren, Bing Li, Zhifen Zhao, Shoudao Li, Wensheng Zhai

**Affiliations:** ^1^The Pediatric Hospital, The First Affiliated Hospital of Henan University of Chinese Medicine, Zhengzhou, China; ^2^College of Pediatrics, Henan University of Chinese Medicine, Zhengzhou, China

**Keywords:** acute kidney injury, chronic kidney disease, gut microbiota, microbial-derived metabolites, fecal microbiota transplantation

## Abstract

Kidney diseases, including acute kidney injury (AKI) and chronic kidney disease (CKD), pose growing global public health challenges. With the emergence and expanding understanding of the “microbiota–gut–kidney axis,” increasing evidence indicates that intestinal barrier disruption, abnormal microbial metabolite production, and intestinal mucosal immune dysregulation play critical roles in the pathogenesis of various kidney diseases. Therapeutic modulation of the gut microbiota through probiotics, prebiotics, synbiotics, and natural products has shown potential for slowing kidney disease progression. Fecal microbiota transplantation (FMT), a direct method of reconstructing gut microbial communities, has demonstrated promise in CKD by targeting mechanisms such as inhibition of the renin–angiotensin system (RAS), attenuation of inflammation and immune activation, and restoration of intestinal barrier integrity. Although FMT has not yet been applied to AKI, its use in CKD subtypes, such as diabetic nephropathy, IgA nephropathy, membranous nephropathy, and focal segmental glomerulosclerosis, has shown encouraging preclinical and preliminary clinical results. This review systematically summarizes the current research on FMT in the context of kidney disease, evaluates its therapeutic mechanisms and feasibility, and highlights its limitations. Most studies remain in the preclinical stage, while available clinical trials are limited by small sample sizes, heterogeneous designs, and lack of standardization. To enhance the translational potential of FMT in nephrology, future studies should incorporate artificial intelligence for personalized intervention strategies and establish standardized protocols to ensure safety, efficacy, and reproducibility.

## 1 Introduction

Kidney diseases, including acute kidney injury (AKI) and chronic kidney disease (CKD), are characterized by abnormalities in kidney function or structure (1, 2). Based on the anatomical regions affected, kidney diseases can be classified into glomerular diseases, tubular disorders, interstitial nephritis, and renal vascular lesions (3–5). AKI commonly occurs in critically ill patients and extremely low birth weight neonates and is often accompanied by multi-organ dysfunction. It is associated with poor in-hospital outcomes (6, 7), increased mortality, and an elevated risk of progression to CKD (8, 9). AKI resulting from glomerular, tubular, and interstitial damage may lead to persistent renal impairment, ultimately advancing into CKD (10, 11). Epidemiological data indicate that the global burden of CKD is increasing, with a reported global prevalence of approximately 10% (12, 13). In China, the Sixth National Chronic Disease and Risk Factor Surveillance reported a CKD prevalence of 8.2% (14). CKD is projected to become the fifth leading cause of death worldwide (15).

Current management strategies for kidney diseases focus on treating the underlying etiology, preventing and managing complications, implementing lifestyle modifications, and controlling risk factors, such as hypertension, hyperglycemia, and dyslipidemia (16–18). Although these interventions offer some therapeutic benefits, limitations persist in achieving optimal clinical outcomes. Therefore, novel therapeutic approaches are urgently required for the prevention and treatment of CKD. In recent years, the concept of the “microbiota–gut–kidney axis” has received increasing attention. Emerging evidence suggests that gut microbiota plays a critical role in the pathogenesis of various kidney diseases (19–22). As such, identifying differences in gut microbial composition between patients with kidney disease and healthy individuals may offer new insights into disease mechanisms and inform future therapeutic strategies.

## 2 The physiological role of gut microbiota

As one of the largest human organs interfacing with the external environment, the gut is colonized by a vast and dense microbial community, constituting the most populous and diverse microbial niche in the human body (23). The surface area of a healthy adult gut is approximately 200 square meters and supports between 500 and 1,000 bacterial species, making it the organ with the greatest microbial abundance and diversity in both quantity and variety (24). The advent of high-throughput next-generation sequencing and other advanced biotechnologies has greatly facilitated systematic characterization of the gut microbiome, including its species composition, relative abundance, community diversity, and functional capacity (25). Although individual microbiota profiles differ owing to factors such as genetics, enterotype, body mass index, exercise frequency, lifestyle, and cultural or dietary habits (26, 27), studies have demonstrated substantial commonality in microbial taxa among individuals (28). Analyses based on bacterial 16S ribosomal RNA (16S rRNA) gene sequencing have indicated that the gut microbiota may include over 160 bacterial species. The dominant phyla are *Bacteroidetes* and *Firmicutes*, which together account for more than 90% of the microbial population, whereas *Proteobacteria* and *Actinobacteria* also constitute major components (25, 29).

Microbial homeostasis in the gut is maintained through a balance between symbiotic and antagonistic interactions between its inhabitants (30, 31). This balance contributes to host health through multiple mechanisms, including nutrient metabolism, immune regulation, and defense against pathogens (32). The primary physiological functions of the gut microbiota include: (1) regulation of nutrient and energy metabolism, aiding in the digestion and absorption of carbohydrates, contributing to the synthesis of amino acids and vitamins, and maintaining essential nutrient balance (33); (2) gut barrier protection, strengthening epithelial tight junctions to preserve mucosal homeostasis, competitively inhibiting pathogen colonization, and mitigating hypersensitivity to food and environmental antigens (34); and (3) production of bioactive metabolites, such as short-chain fatty acids (SCFAs), primarily acetate, propionate, and butyrate (35, 36). A growing body of evidence suggests that SCFAs have therapeutic potential in kidney diseases of various etiologies (37, 38). Other important microbial metabolites include bile acids (39), trimethylamine N-oxide (TMAO) (40), and branched-chain amino acids (41); and (4) modulation of the immune system, which promotes immune cell differentiation, supports immune tolerance, and enhances host defense against pathogens (42, 43).

## 3 The relationship between gut microbiota dysbiosis and kidney disease

The symbiotic relationship between the gut microbiota and host represents a double-edged sword. Although microbiota supports numerous physiological functions, its balance is susceptible to disruption by various internal and external factors. Host genetic background, early-life microbial colonization, dietary habits, smoking, alcohol intake, antibiotic and proton-pump inhibitor use, and underlying disease conditions can all contribute to gut microbiota dysbiosis (43, 44). This ecological imbalance has been implicated in the pathogenesis of multiple diseases, including inflammatory bowel disease (45), obesity (46), CKD (47, 48), atherosclerosis (49), cancer (50, 51), depression (52), and type 2 diabetes (48, 53). In recent years, accumulating evidence has demonstrated that the gut microbiota, through its structural composition, metabolic products, and derived molecules, plays a pivotal regulatory role in the development and progression of various kidney diseases (54–56). Dysbiosis is closely associated with disruption of the intestinal epithelial barrier, altered production of microbial metabolites, and dysregulated intestinal mucosal immune responses, all of which can exert direct detrimental effects on renal function (57).

### 3.1 Disruption of the intestinal barrier

The normal gut microbiota plays a vital role in preserving the structural and functional integrity of the intestinal mucosa. AKI triggers systemic inflammatory responses and fluid overload, which alter the permeability of the mesenteric vascular bed and contribute to intestinal edema, ultimately resulting in secondary damage to the intestinal epithelial barrier (58). Histological analyses of the small intestine following AKI have revealed apoptosis of the deep villous capillary endothelial cells, increased vascular permeability, and epithelial necrosis (59). Tang et al. reported that patients with immunoglobulin A nephropathy (IgAN) exhibit significant gut microbiota dysbiosis and elevated levels of biomarkers indicative of intestinal mucosal barrier injury, including diamine oxidase, soluble intercellular adhesion molecule 1 (sICAM-1), d-lactate, and lipopolysaccharide (LPS) (60). Similarly, in CKD, the intestinal barrier is compromised due to disruption of epithelial tight junction proteins, leading to increased permeability and translocation of bacteria and endotoxins, such as LPS, into the systemic circulation (61). Tang et al. also observed elevated levels of intestinal permeability markers, such as LPS, sICAM-1, and D-lactate, in IgAN mouse models (62). Yang et al. demonstrated that in 5/6 nephrectomized mice, gut microbiota dysbiosis was positively correlated with the severity of intestinal barrier impairment and aberrant mucosal immune responses (63). These findings suggest that disruption of the intestinal barrier may play a critical role in the pathogenesis and progression of CKD (61).

### 3.2 Abnormal production of metabolites

A growing body of evidence has confirmed that kidney diseases are associated with distinct alterations in metabolic profiles, with numerous metabolites being significantly linked to renal function decline (64–69). Gut microbial metabolites have been described as multiple biochemical intermediates (70). Dysbiosis of the gut microbiota can lead to abnormal accumulation of gut-derived uremic toxins such as indoxyl sulfate (IS). Clinical studies have demonstrated that elevated IS levels in patients with AKI are closely associated with poor prognosis. Under pathological conditions, these toxins compromise the intestinal mucosal barrier, exacerbating endotoxemia and systemic inflammation (71).

In CKD, metabolic disturbances impair protein digestion and absorption, contributing to microbial dysbiosis and increased production of protein-derived metabolites, such as p-cresol, indole, phenol, and trimethylamine. These compounds serve as precursors for hepatic synthesis of uremic toxins, including p-cresol sulfate (PCS), IS, phenyl sulfate (PS), and TMAO, which are strongly correlated with deteriorating renal function (72–76). Although partially excreted by the kidneys and intestines, these metabolites exert nephrotoxic effects and are classic uremic toxins. They can activate signaling pathways involved in inflammation and fibrosis, promoting renal inflammation, fibrotic progression, and functional decline (77, 78). The accumulation of uremic toxins can injure renal tubular cells, accelerate glomerulosclerosis and tubulointerstitial fibrosis, and ultimately lead to end-stage renal failure (79).

In addition to protein metabolites, bile acids synthesized from cholesterol via hepatic enzymes also play a role in kidney pathology. This process is regulated by gut microbiota such as *Bacteroides*, *Bifidobacterium*, and *Lactobacillus* (80, 81). Elevated bile acid levels have been identified as an independent risk factor for adverse renal outcomes in diabetic nephropathy (DN) (82). TMAO, which is derived from the microbial degradation of dietary choline and carnitine, is another key metabolite implicated in renal disease. Clinical studies have shown significantly higher TMAO levels in patients with DN than in those with diabetes alone, with a positive correlation between TMAO concentration and the urine protein-to-creatinine ratio (83, 84).

Indole-3-propionic acid, a gut-derived tryptophan metabolite, is significantly reduced in both the gut and serum of patients with IgAN, likely because of the decreased abundance of *Bacteroides* (85). Microbial community profiles also differ across kidney diseases. For example, patients with membranous nephropathy (MN) and IgAN exhibit higher levels of *Megasphaera* and *Bilophila* and lower levels of *Megamonas*, *Veillonella*, *Klebsiella*, and *Streptococcus* than those with MN (86). In end-stage renal disease, nearly 190 operational taxonomic units (OTUs) show altered abundance relative to that in healthy controls (87). Experimental studies have demonstrated that gut microbiota depletion via antibiotic administration reduces TMAO levels and attenuates the transition from AKI to CKD (88). Moreover, supplementation with SCFAs in IgAN mouse models decreased IgA deposition, mesangial proliferation, and proteinuria levels (89). These findings highlight the critical role of gut microbiota dysbiosis and its metabolites in the pathogenesis of kidney disease, highlighting their potential as novel diagnostic biomarkers and therapeutic targets.

## 4 Kidney disease treatment by regulating gut microbiota

Given the close relationship between gut microbiota and the pathogenesis of various kidney diseases, modulation of the gut microbiome has emerged as a promising therapeutic strategy for preventing or slowing disease progression. In this context, the use of microbiota-targeted interventions such as probiotics, prebiotics, synbiotics, and natural products has shown potential in ameliorating renal dysfunction and improving patient outcomes.

### 4.1 Probiotics

Probiotics are live microorganisms that, when administered in adequate amounts, confer health benefits to the host (90). These organisms exert their effects by correcting gut microbial imbalances, producing antimicrobial compounds that inhibit pathogenic bacteria, and enhancing the integrity of the intestinal barrier (90–92). Probiotics also contribute to the restoration of the normal gut pH, suppress the overgrowth of harmful bacteria, promote the production of SCFAs, and maintain gastrointestinal homeostasis.

A clinical study investigating probiotic supplementation in patients with sepsis-induced AKI reported no significant improvement in renal function recovery; however, a downward trend in mortality was observed in the intervention group (93). In a mouse model of ischemia-reperfusion injury (IRI)-induced AKI, Yang et al. demonstrated that *Bifidobacterium bifidum* (BGN4) enhanced microbial evenness and inhibited the proliferation of hallmark AKI-associated taxa, such as *Enterobacteriaceae* and *Bacteroidaceae*. BGN4 administration also significantly reduced neutrophil and macrophage infiltration, and lowered renal interleukin-6 mRNA expression levels. Ikeda et al. identified two novel probiotic strains isolated from fruits and vegetables and found that their supplementation alleviated oxidative stress and AKI by increasing the abundance of *Akkermansia muciniphila* (94). In a study by Miao et al., the taxonomic lineage *Bacilli–Lactobacillales–Lactobacillaceae–Lactobacillus–Lactobacillus johnsonii* were found to be strongly associated with CKD progression, with a significant reduction in *L. johnsonii* abundance observed in rats with adenine-induced CKD. Supplementation with *L. johnsonii* mitigated renal injury (95). The relative abundance of *L. johnsonii* was significantly decreased with progressive CKD in rats with adenine-induced CKD. *L. johnsonii* supplementation attenuates renal damage (95). Ranganathan et al. demonstrated that treatment with *Bacillus pasteurii* and *Lactobacillus sporogenes* effectively slowed CKD progression in a rat model (96). Similarly, Zhou et al. found decreased levels of *Bacteroides fragilis* in both patients with CKD and unilateral ureteral obstruction (UUO) mice. Oral administration of activated *B. fragilis* mitigated renal fibrosis in UUO and adenine-induced models, possibly through mechanisms involving decreased LPS levels and increased concentrations of 1,5-anhydroglucitol (97). Moreover, probiotic therapy has shown beneficial effects in patients undergoing peritoneal dialysis (PD), improving treatment outcomes and offering a potential adjunctive approach in PD management (98). These findings suggest that probiotic supplementation is a promising therapeutic option for kidney disease as it modulates the composition and function of the gut microbiota.

### 4.2 Prebiotics

Prebiotics are defined as non-viable microbial components or substrates selectively utilized by host microorganisms to confer health benefits (99). Compared to live probiotics, prebiotics offer improved stability and safety profiles, making them suitable for various clinical applications (91, 100). These compounds are typically fermentable organic substances that selectively stimulate metabolism and proliferation of beneficial gut bacteria, contributing to host health. Common prebiotics include inulin, fructooligosaccharides (FOS), galactooligosaccharides (GOS), polyphenols, and lactulose (101). While most studies on prebiotics have focused on their effects on CKD, few studies have investigated their role in AKI (101). In a clinical trial by Esgalhado et al., patients with CKD undergoing dialysis were administered resistant starch and compared with a placebo group. The intervention group showed a significant reduction in circulating inflammatory markers and uremic toxins (102). Similarly, in an animal study, CKD rats receiving a diet supplemented with lactose exhibited improved blood urea nitrogen and serum creatinine levels along with reduced tubulointerstitial fibrosis (103).

Multiple studies have demonstrated that prebiotic supplementation can exert renoprotective effects by modulating the gut microbiota composition and restoring intestinal barrier function. This, in turn, helps prevent bacterial translocation and systemic dissemination of harmful microbial metabolites. However, emerging evidence also highlights the potential risks. For instance, a study reported that approximately 40% of TLR5-knockout mice fed a diet containing inulin developed hepatocellular carcinoma, which was associated with a marked increase in *Proteobacteria* and *Clostridium* in the gut microbiota. In contrast, wild-type mice with intact gut microbiota do not develop liver tumors under the same dietary conditions (104). These findings suggest that, while prebiotic intake may improve renal function and inflammation in CKD patients with pre-existing gut dysbiosis, the potential for adverse effects, particularly under conditions of impaired microbial-host immune signaling, warrants careful evaluation and further investigation.

### 4.3 Synbiotics

Synbiotics are defined as combinations of probiotics and prebiotics. Several studies have shown that synbiotic supplementation can positively modulate gut microbiota composition in patients with CKD, including an increase in *Bifidobacterium* and a reduction in *Akkermansia muciniphila* abundance (105, 106). In addition, synbiotics have been reported to reduce serum levels of p-cresol sulfate in both patients with CKD and those undergoing hemodialysis, although they do not appear to significantly affect the serum levels of indoxyl sulfate in CKD patients.

In a clinical trial involving 60 hemodialysis patients, Haghighat et al. demonstrated that synbiotic supplementation significantly reduced serum LPS levels. Moreover, levels of systemic inflammatory markers, including C-reactive protein (CRP), interleukin-6 (IL-6), and anti-heat shock protein 70, were significantly lower in the synbiotic group than in the probiotic and placebo groups (107). These findings suggest that synbiotics may help restore intestinal barrier function, inhibit the overgrowth of gram-negative bacteria, reduce LPS translocation into systemic circulation, alleviate microinflammation, and potentially slow the progression of kidney disease. However, the current evidence on the efficacy of synbiotics in renal disease is limited, and the overall quality and quantity of supporting clinical studies remain relatively low. Further well-designed randomized controlled trials are needed to confirm their therapeutic potential and to establish clinical guidelines for their use in kidney disease management.

### 4.4 Natural products

A growing body of research has demonstrated that natural products exhibit promising clinical efficacy for the treatment of various kidney diseases (108–114). The bioactive components of these natural products can modulate the composition and abundance of the gut microbiota in a holistic manner, alleviating kidney disease progression and renal fibrosis through microbiota-targeted interventions (115–120).

Recent studies have shown that resveratrol significantly reduced serum urea and 24-h urinary protein levels in db/db mice. Additionally, it increases the abundance of beneficial gut bacteria, such as *Bacteroides*, *Lachnospiraceae*, and *Faecalibacterium*, which are associated with anti-inflammatory effects (121). These findings suggest that resveratrol, known for its anti-inflammatory, antioxidant, and anti-glycation properties (122), has therapeutic potential in both AKI (123) and DN treatment (124).

Curcumin, a natural polyphenol and principal renoprotective constituent of turmeric, has also shown beneficial effects (125). In a study by Shi et al., treatment with a docosahexaenoic acid-conjugated curcumin diester significantly reduced the serum levels of blood urea nitrogen, creatinine, LPS, and TMAO in an AKI model. It also decreased malondialdehyde (MDA) concentrations in renal tissues, increased glutathione levels, and altered kidney fatty acid composition, indicating that curcumin effectively suppressed inflammation, oxidative stress, and apoptosis (126). Similarly, Lyu et al. found that astragaloside IV restructured the gut microbiota of DN mice by decreasing the relative abundance of *Firmicutes* and increasing *Bacteroidetes*, *Akkermansia muciniphila*, *Lactobacillus*, *Ligilactobacillus*, *Mucispirillum*, and *Sphaerochaeta*. Conversely, it reduced the abundance of pro-inflammatory taxa such as *Lachnospiraceae*_NK4A136_group, *Lachnospiraceae*, and *Streptococcus*. These microbial changes are associated with decreased LPS levels, improved intestinal mucosal barrier integrity, and reduced renal inflammation (127). In addition, other natural compounds, such as fucoidan (128), peony bark polysaccharide (129), and total alkaloids from mulberry branches (130) have been reported to modulate gut microbiota composition, regulate microbial metabolites, reduce intestinal permeability and systemic inflammation, and attenuate renal pathological damage.

Despite encouraging findings, most current studies on natural products are preclinical and rely heavily on animal models. Few studies have directly correlated microbial changes with renal outcomes in humans. Therefore, future research should emphasize well-designed clinical trials and employ metagenomic or multi-omics approaches to comprehensively elucidate the microbiota-mediated mechanisms by which natural products exert renoprotective effects.

### 4.5 Fecal microbiota transplantation

Fecal microbiota transplantation (FMT) is a therapeutic approach that involves transferring functional gut microbiota from the feces of a healthy donor into the gastrointestinal tract of a recipient via various delivery routes. It is aimed to reconstitute the recipient’s gut microbial community and achieve therapeutic benefits. FMT is considered one of the most direct and effective strategies for restoring gut microbial balance (131, 132). Compared with targeted interventions, such as probiotics, prebiotics, and synbiotics, FMT offers a comprehensive method for eliminating uremic toxins by introducing a diverse and functional microbial ecosystem. Through the introduction of hundreds of commensal microbial species, FMT facilitates intestinal barrier repair, promotes systemic immune modulation, and reestablishes gut–kidney axis homeostasis.

While natural products can exert anti-inflammatory and microbiota-regulating effects via multi-target mechanisms, their clinical application is limited owing to the complex chemical composition and challenges in standardization. In contrast, FMT has shown promise in addressing persistent infections, a major clinical challenge in patients with advanced uremia and those undergoing dialysis. FMT can eliminate multidrug-resistant bacterial colonization through ecological competition, offering long-term control of resistant infections (133). Given its broad-spectrum regulatory capacity, FMT has recently gained attention as a potential therapeutic strategy for the treatment of various kidney diseases. This may represent a promising alternative for protecting renal function by directly modulating the gut microbiota and reducing the inflammatory and toxic burden.

#### 4.5.1 Development and current status of FMT

The concept of FMT dates back to the 17th century, when Italian surgeon Acquapendente reportedly transferred gastrointestinal contents from healthy animals to sick animals, a technique that was later widely adopted in veterinary medicine (134, 135). In the 20th century, FMT was introduced into modern clinical practice, with early reports documenting the use of fecal enemas to treat conditions such as pseudomembranous and ulcerative colitis (136). The early 21st century marked a pivotal moment in the development of FMT. A clinical trial involving the administration of fecal suspension via nasogastric tubes to patients with recurrent *Clostridium difficile* infections (CDI) reported a cure rate of nearly 90% in 18 participants, highlighting FMT as a promising therapeutic approach for CDI (137). In 2013, the first randomized controlled trial of FMT for recurrent CDI was published (138), and later, FMT was officially incorporated into the clinical guidelines for CDI management. The U.S. Food and Drug Administration (FDA) also announced that human feces could be regulated as a drug product, significantly elevating the clinical and regulatory visibility of FMT (139). In 2018, FMT was formally included in the Chinese Consensus on the Diagnosis and Treatment of Inflammatory Bowel Disease, further supporting its clinical application.

In recent years, as research on the gut microbiota has deepened, its role in diverse medical disciplines, including gastroenterology, neurology, immunology, metabolism, and nephrology, has become increasingly evident. FMT, as a potent method for modulating the gut microbiota, has expanded applications across these domains and is progressively demonstrating its therapeutic maturity and translational potential.

#### 4.5.2 Implementation process of FMT

**FIGURE 1:**
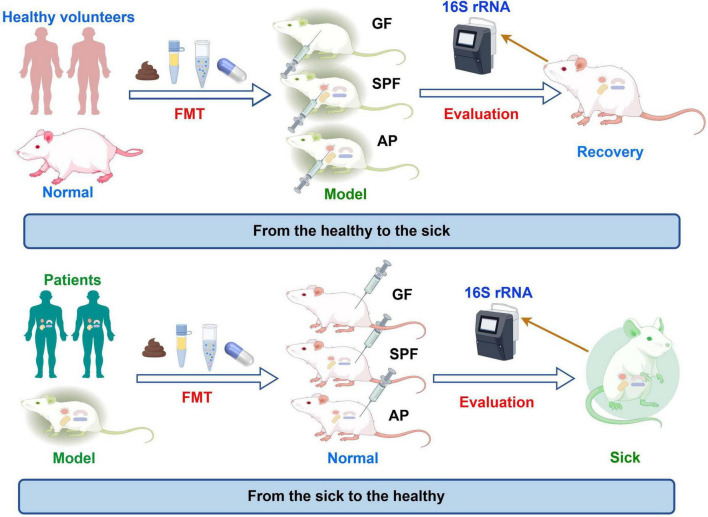
Operation procedure of fecal bacteria transplantation. This figure provides a comprehensive description of the process by which fecal microbiota transplantation (FMT) is applied in animal models for the simulation and treatment of diseases. In the upper half of the figure, fecal samples were obtained from healthy volunteers, and the microbial communities were introduced into germ-free (GF) or specific pathogen-free (SPF) mouse models using FMT technology. An antibiotic pretreated (AP) group was also established to mimic various intestinal environments. Subsequently, changes in microbial communities were evaluated using 16S rRNA gene sequencing, and significant improvements in the health status of the model mice were observed, validating the role of FMT in disease treatment. The lower half of the figure illustrates the process of obtaining fecal samples from patients and introducing their microbial communities into GF or SPF mouse models using FMT, with the establishment of the AP and normal control (normal) groups. Changes in microbial communities were assessed using 16S rRNA gene sequencing, and the manifestation of disease symptoms in the model mice was observed, confirming the potential application of FMT in disease model establishment. Note: The figure was drawn using Figdraw.

The implementation of FMT involves several key steps, including donor and recipient selection, preparation of fecal microbiota suspension, administration of the suspension, and monitoring through gut microbiota analysis. These procedures are essential to ensure the safety, efficacy, and reproducibility of FMT in both the clinical and research settings (Figure 1).

##### 4.5.2.1 Donor selection

To minimize the risk of cross-infection and immune rejection in allogeneic FMT, strict donor screening criteria have been internationally established. According to the Chinese Expert Consensus on the Clinical Application Management of FMT (2022 Edition), donor eligibility is determined by a comprehensive assessment of age, general health status, blood and stool test results, medical history, medication use, psychological status, and gut microbiota profile. Donor sustainability, that is, the ability to repeatedly provide samples over time, is also considered an important selection criterion.

From an ethical and regulatory standpoint, China has more stringent age restrictions than other countries, typically requiring donors to be between 18 and 30 years old. In preclinical and mechanistic studies, fecal material may also be collected from laboratory animals (140), such as rats, mice, or livestock (e.g., cattle, horses, sheep). These animal-derived microbiota samples can be collected from feces or directly from intestinal contents, and are widely used in research on disease pathogenesis and drug development.

##### 4.5.2.2 Recipient selection

Prior to undergoing FMT, human recipients are generally advised to discontinue antibiotic use at least 3 days before the procedure and to undergo bowel cleansing with polyethylene glycol to enhance colonization efficacy (141). In experimental settings, germ-free (GF) mice are commonly used as recipients because of their sterile gastrointestinal environment, which minimizes microbial competition and facilitates the engraftment of donor microbiota (142). However, GF animals have limitations, including high maintenance costs, increased risk of infection, and potential developmental or physiological abnormalities resulting from long-term microbial deprivation. To address these challenges, some studies have utilized animals pretreated with antibiotics or laxatives to partially deplete native gut microbiota and improve the success rate of FMT while reducing the drawbacks associated with GF models.

##### 4.5.2.3 Preparation of fecal microbiota suspension and administration methods

In preparing fecal microbiota suspensions for FMT, studies have shown that there is no significant difference in clinical efficacy between fresh and frozen fecal samples (143). However, repeated freeze–thaw cycles can significantly reduce microbial viability, and consequently, the therapeutic effectiveness of FMT (144). To preserve microbial activity during storage, it is recommended to add 10% glycerol to the fecal suspension and store it at −80°C (145). Given that the gut microbiota is predominantly composed of anaerobic bacteria, the preparation process must be conducted in an anaerobic environment to ensure microbial viability. Fresh fecal samples were promptly transferred to anaerobic containers after collection and transported to the FMT laboratory under controlled conditions.

Common techniques for preparing fecal suspensions include simple filtration, low-speed centrifugation, or a combination of both methods to enrich the microbial content while removing particulate matter (146). In recent years, fecal suspensions have also been formulated into encapsulated preparations for oral use to enhance patient compliance and facilitate administration. In clinical settings, the main routes of FMT administration include upper gastrointestinal tract delivery (via a nasogastric tube or gastroscopy), lower gastrointestinal tract delivery (via colonoscopy or retention enema), and oral capsule administration. To date, no definitive evidence has established the superiority of any single administration route in terms of therapeutic efficacy (147). Therefore, physicians are advised to tailor the route of administration according to each patient’s clinical condition, disease severity, and tolerance. In preclinical animal studies, oral gavage is the most commonly used method for delivering fecal suspensions, whereas rectal administration is employed less frequently.

##### 4.5.2.4 Detection of gut microbiota

In clinical settings, the efficacy of FMT is primarily evaluated based on improvements in clinical symptoms. In basic and translational research, microbial engraftment is typically monitored using molecular techniques such as 16S ribosomal RNA (16S rRNA) sequencing and metagenomic analysis. These approaches allow for a comprehensive assessment of donor microbiota colonization and engraftment, enhancing the reliability and reproducibility of research findings (148). Studies on the duration of microbial engraftment have suggested that the number of donor-derived strains tends to decline over time. While some strains may persist for several months to a few years post-transplantation, most strains demonstrate a gradual decrease in abundance (149). Despite these insights, current data on the long-term persistence and stability of the engrafted microbiota remain limited. Thus, future large-scale longitudinal studies are needed to further clarify the dynamics of microbial colonization and its association with sustained therapeutic efficacy.

#### 4.5.3 Potential molecular mechanisms of FMT on kidney disease

The therapeutic effects of FMT in kidney diseases are mediated by multiple molecular pathways. One of the most critical mechanisms involves modulation of the renin–angiotensin system (RAS), which serves as a vital link between gut microbiota dysbiosis and renal pathology (150, 151). Miao et al. demonstrated that Sirtuin 6 (SIRT6) inhibits the Wnt1/β-catenin signaling pathway, downregulating RAS activity and protecting podocytes from injury (152). In a separate study, FMT significantly ameliorated the premature aging phenotype in SIRT6 knockout mice by reducing inflammation and cellular senescence (153). These findings suggest a potential synergistic effect of FMT and SIRT6 in mitigating renal tissue damage by suppressing RAS activation.

Moreover, gut-derived uremic toxins, such as indoxyl sulfate, p-cresyl sulfate, and TMAO, have been shown to activate RAS, exacerbating renal injury and fibrosis (154). FMT has been reported to reduce circulating levels of these toxins, leading to the attenuation of RAS-mediated fibrotic pathways and subsequent protection of renal function (155, 156). This detoxifying effect is widely recognized as a key mechanism by which FMT exerts renoprotective effects (157). In addition to RAS modulation, FMT contributes to renal protection by restoring immune and metabolic homeostasis in recipients. It alleviates inflammation and corrects metabolic disturbances, slowing progression of kidney damage (158, 159). For instance, Lauriero et al. found that transplantation of healthy human microbiota into an IgAN mouse model reduced renal inflammation and improved glucose tolerance. This effect was attributed to decreased IS levels and increased production of SCFAs which possess anti-inflammatory and renoprotective properties (160). Furthermore, FMT enhances intestinal barrier integrity by downregulating tumor necrosis factor-alpha (TNF-α) expression in intestinal epithelial cells, upregulating tight junction proteins, and reducing LPS translocation. These actions restore intestinal permeability and mitigate systemic inflammation, contributing to the preservation of renal function (161).

Based on current evidence, this review provides a comprehensive summary of the applications of FMT in kidney disease treatment. We highlighted its mechanistic pathways, including RAS inhibition, uremic toxin reduction, metabolic reprogramming, anti-inflammatory effects, and intestinal barrier restoration. We hope that this overview will offer theoretical guidance and support the development of future clinical applications of FMT in nephrology.

## 5 Application of FMT in kidney diseases

FMT is an emerging therapeutic strategy that optimizes the structure and composition of the recipient gut microbiota. By rebalancing microbial communities, FMT reduced the production of gut-derived uremic toxins, mitigated systemic low-grade inflammation, alleviated renal injury, and slowed the progression of CKD (162). This approach has demonstrated potential in the treatment of various kidney diseases.

### 5.1 Diabetic nephropathy

Diabetic nephropathy (DN) is one of the most common microvascular complications of diabetes and is characterized by a range of pathological changes, including mesangial matrix expansion, excessive extracellular matrix deposition, podocyte effacement, glomerulosclerosis, and tubulointerstitial fibrosis, largely driven by persistent hyperglycemia (163). Accumulating evidence indicates that the gut microbiota of patients with DN is significantly altered (164).

Proteinuria is a hallmark of DN. One study demonstrated that differences in gut microbiota might influence renal function in DN mouse models depending on the sequence of FMT and streptozotocin (STZ) administration (133). In this study, severe proteinuria (SP) and mild proteinuria (MP) mouse models were established via intraperitoneal injection of STZ. Microbiota analysis revealed that the *Firmicutes/Bacteroidetes* ratio was higher in the MP group than that in the SP group. At the genus level, *Allobaculum* and *Anaerosporobacter* were enriched in the SP group, whereas *Blautia* was more abundant in the MP group.

FMT experiments have also demonstrated that inulin-type fructans (ITFs) may prevent the development of DN by modifying the gut microbial composition and enhancing SCFA production, as confirmed by FMT-based verification (165). Similarly, Lu et al. reported that FMT from healthy donors significantly improved podocyte insulin sensitivity, alleviated glomerular injury, and reduced proteinuria in DN rats (166). Shang et al. conducted *in vivo* experiments in which DN mice were first treated with broad-spectrum antibiotics to eliminate endogenous microbiota, followed by FMT in healthy donors. The study found significant differences in fecal microbiota composition between the FMT group and the untreated DN model group, confirming that FMT can modulate microbial communities and improve the metabolic phenotype of DN mice (167). Additionally, Cai et al. transplanted fecal microbiota from resveratrol-treated donors into db/db mice and found that FMT not only restored the gut microbial balance but also significantly reduced inflammatory responses (121). This result further supports the role of the microbiota–gut–kidney axis in the protective effects of resveratrol against DN. Similarly, a study involving astragaloside IV (AS-IV) demonstrated that FMT using microbiota from AS-IV-treated donors reshaped gut microbial composition, improved intestinal permeability, and attenuated renal dysfunction in db/db mice (127). Although numerous animal studies have confirmed the beneficial effects of FMT in DN models, clinical trials are scarce. Therefore, further research, particularly well-designed human studies, are warranted to explore the clinical applicability of FMT in DN treatment.

### 5.2 IgA nephropathy

Although the precise etiology and pathogenesis of IgAN remain incompletely understood, accumulating evidence has revealed a strong association between gut microbiota dysbiosis and the development and progression of the disease (168). In one study, fecal, urinary, and serum samples from patients with IgAN were analyzed and compared with those of healthy controls, revealing marked differences in gut microbial composition and associated metabolites (169).

Zhao et al. reported the first case study on the use of FMT in two patients with refractory IgAN unresponsive to immunosuppressive therapy (170). The patients underwent regular FMT via an endoscopic intestinal tube over a 6–7 month period. Follow-up results showed that 24-h urinary protein excretion was reduced to less than 50% of the baseline values, achieving partial clinical remission without any adverse events. Prior to treatment, both patients exhibited reduced microbial diversity and altered gut microbiota composition, which were significantly corrected after FMT. Similarly, Zhi et al. described a case of IgAN in which oral administration of fecal microbiota capsules led to clinical symptom improvement. A six-month follow-up revealed no serious adverse events (171). To further assess the safety and efficacy of FMT in IgAN, Zhi et al. conducted a clinical observational study involving 15 patients (172). Urinary protein levels, gut microbiota profiles, and fecal metabolomic data were analyzed before and after FMT. The study found significant alterations in microbial composition and metabolites. The relative abundances of *Phocaeicola_vulgatus, Bacteroides_uniformis,Prevotella_copri*,*Parabacteroides_distasonis, Phocaeicola_dorei,Bacteroides_sp._HF-162, Bacteroides_ovatus, Bacteroides_xylanisolvens, Bifidobacterium_pseudocatenulatum* and *Bifidobacterium_longum* changed after FMT, indicating successful microbiota reconstruction and suggesting a link between these changes and improved renal function.

In mechanistic studies, Zhu et al. demonstrated that gut microbiota dysbiosis can stimulate the overproduction of galactose-deficient IgA1 (Gd-IgA1), a key pathogenic molecule in IgAN, via the Toll-like receptor 4 (TLR4) signaling and B-cell activation pathways (173). Lauriero et al. further observed elevated levels of Gd-IgA1 and serum B-cell-activating factor in patients with IgAN. In an IgAN mouse model, FMT from healthy human donors significantly reduced proteinuria and renal inflammation (160). These findings suggest that reshaping gut microbiota through FMT may modulate immune responses and renal injury in IgAN.

These studies highlight the therapeutic potential of FMT in IgAN by restoring the gut microbial balance, altering metabolite profiles, and modulating key pathogenic pathways. However, further clinical trials are needed to establish the efficacy, safety, and standardized treatment protocols for FMT in IgAN management.

### 5.3 Membranous nephropathy

Membranous nephropathy is the most common pathological subtype of nephrotic syndrome among adults. It is primarily characterized by the deposition of immune complexes on the outer aspect of the glomerular basement membrane, leading to diffuse thickening (174). The standard treatment strategies for MN include supportive care, corticosteroids, and immunosuppressive agents (174). In recent years, increasing attention has been given to the gut–kidney axis in MN, with studies revealing significant differences in gut microbiota composition between patients with MN and healthy individuals (175, 176). Shang et al. analyzed 825 fecal samples collected from patients with MN and healthy controls across Central, East, and South China using 16S rRNA gene sequencing. The study reported markedly reduced microbial diversity and richness in MN patients compared to healthy individuals, and subsequently developed a non-invasive diagnostic model based on these microbial differences (177). Furthermore, the role of gut microbiota in MN pathogenesis was investigated using a rat model. Elimination of the gut microbiota in MN model rats prevented disease onset, whereas FMT restored the proteinuria phenotype, suggesting a causal role of gut dysbiosis in MN development. In a related study, Shi et al. collected fecal samples from 82 individuals with idiopathic MN and healthy volunteers. They identified 20 characteristic microbial biomarkers that were significantly correlated with the clinical features of MN and constructed a predictive diagnostic model with an accuracy of 93.53%. FMT experiments in MN model mice showed that dysbiosis leads to impaired intestinal permeability and activation of renal NOD-like receptors, contributing to MN pathogenesis (175). Zhou et al. reported a clinical case of successful MN treatment using FMT (178). After stringent donor screening, fecal microbiota were obtained from a 14-year-old male donor and prepared for transplantation. The patient underwent two FMT procedures 1 month apart. Following treatment, improvements were observed in serum albumin and total protein levels, and 24-h urinary protein excretion significantly reduced. A transient low-grade fever occurred after the first FMT, but resolved spontaneously, suggesting a generally favorable safety profile.

While these findings indicate the potential of FMT as a novel biological therapy for MN, further validation is necessary. Large-scale clinical trials and mechanistic studies are needed to better establish the therapeutic efficacy, mechanisms, and safety of FMT for MN management.

### 5.4 Focal segmental glomerulosclerosis

Focal segmental glomerulosclerosis (FSGS) is a common and treatment-resistant form of nephrotic syndrome, characterized by effacement of podocyte foot processes and, under electron microscopy, thickening of the glomerular basement membrane and mesangial expansion in sclerotic regions. Zhi et al. reported a case in which FMT using fecal microbiota capsules led to clinical improvement in a patient with FSGS (179). The patient had previously required glucocorticoid maintenance to control serum creatinine levels. Following FMT, renal function remained stable despite glucocorticoid tapering, and reductions were observed in proteinuria and triglyceride and cholesterol levels, ultimately achieving complete clinical remission. This case suggests that FMT may serve as a potential adjuvant therapy for FSGS by reconstituting the gut microbiota to improve renal function and prevent metabolic abnormalities. However, no randomized controlled trials have defined the specific or long-term efficacy of FMT for FSGS. Therefore, further clinical research is essential to evaluate its safety, therapeutic value, and mechanisms of action in this context.

## 6 Limitations and future perspectives of FMT in kidney diseases

### 6.1 Limitations of FMT in kidney diseases

Although FMT represents an innovative therapeutic strategy for kidney disease, its application remains largely confined to preclinical research. Existing clinical trials are limited by small sample sizes and short follow-up periods, and the long-term efficacy and safety of FMT in larger patient populations have yet to be fully established.

#### 6.1.1 Limited clinical evidence

As an emerging treatment for kidney diseases, current clinical studies on FMT are generally limited by their small sample sizes and short follow-up durations. Consequently, the long-term benefits of FMT in larger patient populations remain unclear.

#### 6.1.2 Insufficient monitoring of microbiota stability

Most current studies do not adequately monitor the stability of gut microbiota following FMT. It is recommended that follow-up assessments extend for at least 4 weeks and, when feasible, incorporate microbiomic analyses to dynamically track changes in microbial composition and function.

#### 6.1.3 Unexplored diseases

The pathogenesis of certain kidney diseases, such as lupus nephritis, Henoch-Schönlein purpura nephritis, and sepsis-associated acute kidney injury, has been proven to be related to the gut microbiota. However, research on FMT in these diseases is lacking.

#### 6.1.4 Limited evaluation of adverse effects

Current studies on the adverse effects of FMT are limited. Future studies should strengthen the assessment of these effects and develop scientific treatment guidelines to standardize the risk management of FMT, balancing its therapeutic benefits and potential risks.

### 6.2 Future perspectives

With ongoing advances in biological research, studies investigating the role of FMT in kidney diseases, particularly through modulation of the “microbiota–gut–kidney axis,” are becoming increasingly comprehensive. Strengthening research on gut microbiota is critical for the prevention and treatment of kidney diseases. Thus, the application of FMT in this field holds considerable promise. Future research directions may include the following.

#### 6.2.1 Integration with AI

Leveraging AI technologies may enable the development of personalized FMT treatment strategies, optimize donor–recipient matching, streamline implementation protocols, and enhance post-transplantation monitoring. However, practical frameworks for integrating AI into FMT workflows remain to be established and warrant further investigation.

#### 6.2.2 Specific microbiota donors

Emerging evidence suggests that certain microbial strains in the gut exert disease-specific therapeutic effects. Future research may explore whether individuals with distinctive microbiota profiles beyond those of healthy donors could serve as optimized donors for targeted FMT, enhancing therapeutic outcomes in specific kidney disease subtypes.

#### 6.2.3 Dietary interventions

Diet is one of the most direct and influential factors affecting the composition of the gut microbiota. Future studies should investigate whether specific dietary interventions can support the engraftment of donor microbiota following FMT and modulate microbial metabolism to sustain therapeutic efficacy in kidney disease management.

#### 6.2.4 Ethical and legal considerations

As a form of “organoid transplantation,” FMT raises important ethical and legal concerns. Future efforts should ensure rigorous compliance with donor screening and processing standards, while safeguarding the privacy and informed consent of both donors and recipients.

## References

[1] Francis A Harhay M Ong A Tummalapalli S Ortiz A Fogo A Chronic kidney disease and the global public health agenda: An international consensus. *Nat Rev Nephrol.* (2024) 20:473–85. 10.1038/s41581-024-00820-6 38570631

[2] Cao F Li Y Peng T Li Y Yang L Hu L PTEN in kidney diseases: A potential therapeutic target in preventing AKI-to-CKD transition. *Front Med (Lausanne).* (2024) 11:1428995. 10.3389/fmed.2024.1428995 39165377 PMC11333338

[3] Meliambro K He J Campbell K. Podocyte-targeted therapies - Progress and future directions. *Nat Rev Nephrol.* (2024) 20:643–58. 10.1038/s41581-024-00843-z 38724717

[4] Huang R Fu P Ma L. Kidney fibrosis: From mechanisms to therapeutic medicines. *Signal Transduct Target Ther.* (2023) 8:129. 10.1038/s41392-023-01379-7 36932062 PMC10023808

[5] Amatruda M Carucci N Chimenz R Conti G. Immunoglobulin A vasculitis nephritis: Current understanding of pathogenesis and treatment. *World J Nephrol.* (2023) 12:82–92. 10.5527/wjn.v12.i4.82 37766840 PMC10520755

[6] Lee S Cozzi M Bush E Rabb H. Distant organ dysfunction in acute kidney injury: A review. *Am J Kidney Dis.* (2018) 72:846–56. 10.1053/j.ajkd.2018.03.028 29866457 PMC6252108

[7] Zarbock A Forni L Ostermann M Ronco C Bagshaw S Mehta R Designing acute kidney injury clinical trials. *Nat Rev Nephrol.* (2024) 20:137–46. 10.1038/s41581-023-00758-1 37653237

[8] Starr M Griffin R Harer M Soranno D Gist K Segar J Acute kidney injury defined by fluid-corrected creatinine in premature neonates: A secondary analysis of the PENUT randomized clinical trial. *JAMA Netw Open.* (2023) 6:e2328182. 10.1001/jamanetworkopen.2023.28182 37561461 PMC10415963

[9] Li C Yu B Gao Q Dong H Li Z. The critical role of ion channels in kidney disease: Perspective from AKI and CKD. *Ren Fail.* (2025) 47:2488139. 10.1080/0886022X.2025.2488139 40289808 PMC12039425

[10] Allinson C Pollock C Chen X. Mesenchymal stem cells in the treatment of acute kidney injury (AKI), chronic kidney disease (CKD) and the AKI-to-CKD transition. *Integr Med Nephrol Androl.* (2023) 10:e00014. 10.1097/imna-d-22-00014

[11] Zhang T Widdop R Ricardo S. Transition from acute kidney injury to chronic kidney disease: Mechanisms, models, and biomarkers. *Am J Physiol Renal Physiol.* (2024) 327:F788–805. 10.1152/ajprenal.00184.2024 39298548

[12] Sundström J Bodegard J Bollmann A Vervloet M Mark P Karasik A Prevalence, outcomes, and cost of chronic kidney disease in a contemporary population of 2.4 million patients from 11 countries: The CaReMe CKD study. *Lancet Reg Health Eur.* (2022) 20:100438. 10.1016/j.lanepe.2022.100438 36090671 PMC9459126

[13] Khandpur S Mishra P Mishra S Tiwari S. Challenges in predictive modelling of chronic kidney disease: A narrative review. *World J Nephrol.* (2024) 13:97214. 10.5527/wjn.v13.i3.97214 39351189 PMC11439095

[14] Wang L Xu X Zhang M Hu C Zhang X Li C Prevalence of chronic kidney disease in China: Results from the sixth china chronic disease and risk factor surveillance. *JAMA Intern Med.* (2023) 183:298–310. 10.1001/jamainternmed.2022.6817 36804760 PMC9941971

[15] Fontecha-Barriuso M Lopez-Diaz A Guerrero-Mauvecin J Miguel V Ramos A Sanchez-Niño M Tubular mitochondrial dysfunction, oxidative stress, and progression of chronic kidney disease. *Antioxidants (Basel).* (2022) 11:1356. 10.3390/antiox11071356 35883847 PMC9311633

[16] Floege J Gibson K Vivarelli M Liew A Radhakrishnan J Balk E Executive summary of the KDIGO 2025 clinical practice guideline for the management of nephrotic syndrome in children. *Kidney Int.* (2025) 107:806–8. 10.1016/j.kint.2024.11.006 40254362

[17] Fried L Emanuele N Zhang J Brophy M Conner T Duckworth W Combined angiotensin inhibition for the treatment of diabetic nephropathy. *N Engl J Med.* (2013) 369:1892–903. 10.1056/NEJMoa1303154 24206457

[18] Raikou V. Renoprotective strategies. *World J Nephrol.* (2024) 13:89637. 10.5527/wjn.v13.i1.89637 38596266 PMC11000037

[19] Tao P Huo J Chen L. Bibliometric analysis of the relationship between gut microbiota and chronic kidney disease from 2001–2022. *Integr Med Nephrol Androl.* (2024) 11:e00017. 10.1097/imna-d-23-00017

[20] Tian E Wang F Zhao L Sun Y Yang J. The pathogenic role of intestinal flora metabolites in diabetic nephropathy. *Front Physiol.* (2023) 14:1231621. 10.3389/fphys.2023.1231621 37469558 PMC10352811

[21] Chu C Behera T Huang Y Qiu W Chen J Shen Q. Research progress of gut microbiome and diabetic nephropathy. *Front Med (Lausanne).* (2024) 11:1490314. 10.3389/fmed.2024.1490314 39735707 PMC11671260

[22] Kim M Cho W Chung S Choi Y Fang Y Park M Altered gut microbiome plays an important role in AKI to CKD transition in aged mice. *Front Med (Lausanne).* (2023) 10:1238960. 10.3389/fmed.2023.1238960 38020091 PMC10644820

[23] Kundu P Blacher E Elinav E Pettersson S. Our gut microbiome: The evolving inner self. *Cell.* (2017) 171:1481–93. 10.1016/j.cell.2017.11.024 29245010

[24] Lin K Zhu L Yang L. Gut and obesity/metabolic disease: Focus on microbiota metabolites. *MedComm.* (2020) 2022:e171. 10.1002/mco2.171 36092861 PMC9437302

[25] Qin J Li R Raes J Arumugam M Burgdorf K Manichanh C A human gut microbial gene catalogue established by metagenomic sequencing. *Nature.* (2010) 464:59–65. 10.1038/nature08821 20203603 PMC3779803

[26] Rinninella E Raoul P Cintoni M Franceschi F Miggiano G Gasbarrini A What is the healthy gut microbiota composition? A changing ecosystem across age, environment, diet, and diseases. *Microorganisms.* (2019) 7:14. 10.3390/microorganisms7010014 30634578 PMC6351938

[27] Illiano P Brambilla R Parolini C. The mutual interplay of gut microbiota, diet and human disease. *FEBS J.* (2020) 287:833–55. 10.1111/febs.15217 31955527

[28] Arumugam M Raes J Pelletier E Le Paslier D Yamada T Mende D Enterotypes of the human gut microbiome. *Nature.* (2011) 473:174–80. 10.1038/nature09944 21508958 PMC3728647

[29] Ley R Peterson D Gordon J. Ecological and evolutionary forces shaping microbial diversity in the human intestine. *Cell.* (2006) 124:837–48. 10.1016/j.cell.2006.02.017 16497592

[30] Bäckhed F Ley R Sonnenburg J Peterson D Gordon J. Host-bacterial mutualism in the human intestine. *Science.* (2005) 307:1915–20. 10.1126/science.1104816 15790844

[31] Valdes A Walter J Segal E Spector T. Role of the gut microbiota in nutrition and health. *BMJ.* (2018) 361:k2179. 10.1136/bmj.k2179 29899036 PMC6000740

[32] Adak A Khan M. An insight into gut microbiota and its functionalities. *Cell Mol Life Sci.* (2019) 76:473–93. 10.1007/s00018-018-2943-4 30317530 PMC11105460

[33] Hooper L Midtvedt T Gordon J. How host-microbial interactions shape the nutrient environment of the mammalian intestine. *Annu Rev Nutr.* (2002) 22:283–307. 10.1146/annurev.nutr.22.011602.092259 12055347

[34] Alam A Neish A. Role of gut microbiota in intestinal wound healing and barrier function. *Tissue Barriers.* (2018) 6:1539595. 10.1080/21688370.2018.1539595 30404570 PMC6389125

[35] Li Y Chen X Kwan T Loh Y Singer J Liu Y Dietary fiber protects against diabetic nephropathy through short-chain fatty acid-mediated activation of G protein-coupled receptors GPR43 and GPR109A. *J Am Soc Nephrol.* (2020) 31:1267–81. 10.1681/ASN.2019101029 32358041 PMC7269358

[36] Martin-Gallausiaux C Marinelli L Blottière H Larraufie P Lapaque N. SCFA: Mechanisms and functional importance in the gut. *Proc Nutr Soc.* (2021) 80:37–49. 10.1017/S0029665120006916 32238208

[37] Yang J Dong H Wang Y Jiang Y Zhang W Lu Y Cordyceps cicadae polysaccharides ameliorated renal interstitial fibrosis in diabetic nephropathy rats by repressing inflammation and modulating gut microbiota dysbiosis. *Int J Biol Macromol.* (2020) 163:442–56. 10.1016/j.ijbiomac.2020.06.153 32592781

[38] Rooks M Garrett W. Gut microbiota, metabolites and host immunity. *Nat Rev Immunol.* (2016) 16:341–52. 10.1038/nri.2016.42 27231050 PMC5541232

[39] Cai J Sun L Gonzalez F. Gut microbiota-derived bile acids in intestinal immunity, inflammation, and tumorigenesis. *Cell Host Microbe.* (2022) 30:289–300. 10.1016/j.chom.2022.02.004 35271802 PMC8923532

[40] Gatarek P Kaluzna-Czaplinska J. Trimethylamine N-oxide (TMAO) in human health. *EXCLI J.* (2021) 20:301–19. 10.17179/excli2020-3239 33746664 PMC7975634

[41] Zhang Y He X Qian Y Xu S Mo C Yan Z Plasma branched-chain and aromatic amino acids correlate with the gut microbiota and severity of Parkinson’s disease. *NPJ Parkinsons Dis.* (2022) 8:48. 10.1038/s41531-022-00312-z 35449203 PMC9023571

[42] Yang R Chen Z Cai J. Fecal microbiota transplantation: Emerging applications in autoimmune diseases. *J Autoimmun.* (2023) 141:103038. 10.1016/j.jaut.2023.103038 37117118

[43] Fan L Xia Y Wang Y Han D Liu Y Li J Gut microbiota bridges dietary nutrients and host immunity. *Sci China Life Sci.* (2023) 66:2466–514. 10.1007/s11427-023-2346-1 37286860 PMC10247344

[44] David L Maurice C Carmody R Gootenberg D Button J Wolfe B Diet rapidly and reproducibly alters the human gut microbiome. *Nature.* (2014) 505:559–63. 10.1038/nature12820 24336217 PMC3957428

[45] Pan Y Zhang H Li M He T Guo S Zhu L Novel approaches in IBD therapy: Targeting the gut microbiota-bile acid axis. *Gut Microbes.* (2024) 16:2356284. 10.1080/19490976.2024.2356284 38769683 PMC11110704

[46] Liu B Liu X Liang Z Wang J. Gut microbiota in obesity. *World J Gastroenterol.* (2021) 27:3837–50. 10.3748/wjg.v27.i25.3837 34321848 PMC8291023

[47] Cooper T Khalid R Chan S Craig J Hawley C Howell M Synbiotics, prebiotics and probiotics for people with chronic kidney disease. *Cochrane Database Syst Rev.* (2023) 10:CD013631. 10.1002/14651858.CD013631.pub2 37870148 PMC10591284

[48] Yang T Richards E Pepine C Raizada M. The gut microbiota and the brain-gut-kidney axis in hypertension and chronic kidney disease. *Nat Rev Nephrol.* (2018) 14:442–56. 10.1038/s41581-018-0018-2 29760448 PMC6385605

[49] Witkowski M Weeks T Hazen S. Gut microbiota and cardiovascular disease. *Circ Res.* (2020) 127:553–70. 10.1161/CIRCRESAHA.120.316242 32762536 PMC7416843

[50] Wang Z Dan W Zhang N Fang J Yang Y. Colorectal cancer and gut microbiota studies in China. *Gut Microbes.* (2023) 15:2236364. 10.1080/19490976.2023.2236364 37482657 PMC10364665

[51] Qu R Zhang Y Ma Y Zhou X Sun L Jiang C Role of the gut microbiota and its metabolites in tumorigenesis or development of colorectal cancer. *Adv Sci (Weinh).* (2023) 10:e2205563. 10.1002/advs.202205563 37263983 PMC10427379

[52] Liu L Wang H Chen X Zhang Y Zhang H Xie P. Gut microbiota and its metabolites in depression: From pathogenesis to treatment. *EBioMedicine.* (2023) 90:104527. 10.1016/j.ebiom.2023.104527 36963238 PMC10051028

[53] Wu J Yang K Fan H Wei M Xiong Q. Targeting the gut microbiota and its metabolites for type 2 diabetes mellitus. *Front Endocrinol (Lausanne).* (2023) 14:1114424. 10.3389/fendo.2023.1114424 37229456 PMC10204722

[54] Wang X Yang S Li S Zhao L Hao Y Qin J Aberrant gut microbiota alters host metabolome and impacts renal failure in humans and rodents. *Gut.* (2020) 69:2131–42. 10.1136/gutjnl-2019-319766 32241904 PMC7677483

[55] Antza C Stabouli S Kotsis V. Gut microbiota in kidney disease and hypertension. *Pharmacol Res.* (2018) 130:198–203. 10.1016/j.phrs.2018.02.028 29496593

[56] Yao K Zheng L Chen W Xie Y Liao C Zhou T. Characteristics, pathogenic and therapeutic role of gut microbiota in immunoglobulin A nephropathy. *Front Immunol.* (2025) 16:1438683. 10.3389/fimmu.2025.1438683 39981255 PMC11839611

[57] Chi M Ma K Wang J Ding Z Li Y Zhu S The immunomodulatory effect of the gut microbiota in kidney disease. *J Immunol Res.* (2021) 2021:5516035. 10.1155/2021/5516035 34095319 PMC8140847

[58] Gong J Noel S Pluznick J Hamad A Rabb H. Gut microbiota-kidney cross-talk in acute kidney injury. *Semin Nephrol.* (2019) 39:107–16. 10.1016/j.semnephrol.2018.10.009 30606403 PMC6322425

[59] Park S Chen S Kim M Brown K Kolls J D’Agati V Cytokines induce small intestine and liver injury after renal ischemia or nephrectomy. *Lab Invest.* (2011) 91:63–84. 10.1038/labinvest.2010.151 20697374 PMC2991383

[60] Tang Y Zhu Y He H Peng Y Hu P Wu J Gut dysbiosis and intestinal barrier dysfunction promotes IgA nephropathy by increasing the production of Gd-IgA1. *Front Med (Lausanne).* (2022) 9:944027. 10.3389/fmed.2022.944027 35872757 PMC9302483

[61] Vaziri N Yuan J Rahimi A Ni Z Said H Subramanian V. Disintegration of colonic epithelial tight junction in uremia: A likely cause of CKD-associated inflammation. *Nephrol Dial Transplant.* (2012) 27:2686–93. 10.1093/ndt/gfr624 22131233 PMC3616758

[62] Tang Y Xiao Y He H Zhu Y Sun W Hu P Aberrant gut microbiome contributes to barrier dysfunction, inflammation, and local immune responses in IgA nephropathy. *Kidney Blood Press Res.* (2023) 48:261–76. 10.1159/000528973 36878203 PMC10158088

[63] Yang J Lim S Ko Y Lee H Oh S Kim M Intestinal barrier disruption and dysregulated mucosal immunity contribute to kidney fibrosis in chronic kidney disease. *Nephrol Dial Transplant.* (2019) 34:419–28. 10.1093/ndt/gfy172 29939312

[64] Gan L Wang L Li W Zhang Y Xu B. Metabolomic profile of secondary hyperparathyroidism in patients with chronic kidney disease stages 3-5 not receiving dialysis. *Front Endocrinol (Lausanne).* (2024) 15:1406690. 10.3389/fendo.2024.1406690 39027473 PMC11254665

[65] Zhang W Zheng J Zhang J Li N Yang X Fang Z Associations of serum amino acids related to urea cycle with risk of chronic kidney disease in Chinese with type 2 diabetes. *Front Endocrinol (Lausanne).* (2023) 14:1117308. 10.3389/fendo.2023.1117308 36936143 PMC10018121

[66] Wang Y Zhang Z Liu H Guo Z Zou L Zhang Y Integrative phosphatidylcholine metabolism through phospholipase A2 in rats with chronic kidney disease. *Acta Pharmacol Sin.* (2023) 44:393–405. 10.1038/s41401-022-00947-x 35922553 PMC9889763

[67] Lin W Mousavi F Blum B Heckendorf C Moore J Lampl N Integrated metabolomics and proteomics reveal biomarkers associated with hemodialysis in end-stage kidney disease. *Front Pharmacol.* (2023) 14:1243505. 10.3389/fphar.2023.1243505 38089059 PMC10715419

[68] Tan R Ou S Kang T Wu W Xiong L Zhu T Altered serum metabolome associated with vascular calcification developed from CKD and the critical pathways. *Front Cardiovasc Med.* (2023) 10:1114528. 10.3389/fcvm.2023.1114528 37113701 PMC10126378

[69] Chen D Guo Y Li P. New insights into a novel metabolic biomarker and therapeutic target for chronic kidney disease. *Integr Med Nephrol Androl.* (2024) 11:e24–00019. 10.1097/imna-d-24-00019

[70] Krautkramer K Fan J Bäckhed F. Gut microbial metabolites as multi-kingdom intermediates. *Nat Rev Microbiol.* (2021) 19:77–94. 10.1038/s41579-020-0438-4 32968241

[71] Wang W Hao G Pan Y Ma S Yang T Shi P Serum indoxyl sulfate is associated with mortality in hospital-acquired acute kidney injury: A prospective cohort study. *BMC Nephrol.* (2019) 20:57. 10.1186/s12882-019-1238-9 30764800 PMC6376694

[72] Ravid J Kamel M Chitalia V. Uraemic solutes as therapeutic targets in CKD-associated cardiovascular disease. *Nat Rev Nephrol.* (2021) 17:402–16. 10.1038/s41581-021-00408-4 33758363

[73] Corradi V Caprara C Barzon E Mattarollo C Zanetti F Ferrari F A possible role of p-cresyl sulfate and indoxyl sulfate as biomarkers in the prediction of renal function according to the GFR (G) categories. *Integr Med Nephrol Androl.* (2024) 11:e24–00002. 10.1097/imna-d-24-00002

[74] Zeisel S Warrier M. Trimethylamine N-Oxide, the microbiome, and heart and kidney disease. *Annu Rev Nutr.* (2017) 37:157–81. 10.1146/annurev-nutr-071816-064732 28715991

[75] Zhao B Hu X Wang W Zhou Y. Cardiorenal syndrome: Clinical diagnosis, molecular mechanisms and therapeutic strategies. *Acta Pharmacol Sin.* (2025) 46:1539–55. 10.1038/s41401-025-01476-z 39910210 PMC12098865

[76] Cao G Miao H Wang Y Chen D Wu X Chen L Intrarenal 1-methoxypyrene, an aryl hydrocarbon receptor agonist, mediates progressive tubulointerstitial fibrosis in mice. *Acta Pharmacol Sin.* (2022) 43:2929–45. 10.1038/s41401-022-00914-6 35577910 PMC9622813

[77] Vaziri N. Effect of synbiotic therapy on gut-derived uremic toxins and the intestinal microbiome in patients with CKD. *Clin J Am Soc Nephrol.* (2016) 11:199–201. 10.2215/CJN.13631215 26772192 PMC4741052

[78] Li X Shan Q Wu X Miao H Zhao Y. Gut microbiota regulates oxidative stress and inflammation: A double-edged sword in renal fibrosis. *Cell Mol Life Sci.* (2024) 81:480. 10.1007/s00018-024-05532-5 39636415 PMC11621299

[79] Chao C Lin S. Uremic toxins and frailty in patients with chronic kidney disease: A molecular insight. *Int J Mol Sci.* (2021) 22:6270. 10.3390/ijms22126270 34200937 PMC8230495

[80] Zhang Q Lu L Wang J Lu M Liu D Zhou C Metabolomic profiling reveals the step-wise alteration of bile acid metabolism in patients with diabetic kidney disease. *Nutr Diabetes.* (2024) 14:85. 10.1038/s41387-024-00315-0 39384774 PMC11464666

[81] Li X Fang C Zhao R Zou L Miao H Zhao Y. Bile acid metabolism in health and ageing-related diseases. *Biochem Pharmacol.* (2024) 225:116313. 10.1016/j.bcp.2024.116313 38788963

[82] Xiao X Zhang J Ji S Qin C Wu Y Zou Y Lower bile acids as an independent risk factor for renal outcomes in patients with type 2 diabetes mellitus and biopsy-proven diabetic kidney disease. *Front Endocrinol (Lausanne).* (2022) 13:1026995. 10.3389/fendo.2022.1026995 36277729 PMC9585231

[83] Yang M Zhang R Zhuang C Wu Y Yang Q Yu Z Serum trimethylamine n-oxide and the diversity of the intestinal microbial flora in Type 2 diabetes complicated by diabetic kidney disease. *Clin Lab.* (2022) 68:210836. 10.7754/Clin.Lab.2021.210836 35536069

[84] Huang Y Zhu Z Huang Z Zhou J. Elevated serum trimethylamine oxide levels as potential biomarker for diabetic kidney disease. *Endocr Connect.* (2023) 12:e220542. 10.1530/EC-22-0542 37183928 PMC10388659

[85] Wu H Tang D Yun M Liu H Huang S Yun C Metabolic dysfunctions of intestinal fatty acids and tryptophan reveal immuno-inflammatory response activation in IgA nephropathy. *Front Med (Lausanne).* (2022) 9:811526. 10.3389/fmed.2022.811526 35186998 PMC8850467

[86] Dong R Bai M Zhao J Wang D Ning X Sun SA. Comparative study of the gut microbiota associated with immunoglobulin a nephropathy and membranous nephropathy. *Front Cell Infect Microbiol.* (2020) 10:557368. 10.3389/fcimb.2020.557368 33194798 PMC7606180

[87] Vaziri N Wong J Pahl M Piceno Y Yuan J DeSantis T Chronic kidney disease alters intestinal microbial flora. *Kidney Int.* (2013) 83:308–15. 10.1038/ki.2012.345 22992469

[88] Lee J Lee J Kim K Lee J Jung Y Hyeon J Antibiotic-induced intestinal microbiota depletion can attenuate the acute kidney injury to chronic kidney disease transition via NADPH oxidase 2 and trimethylamine-N-oxide inhibition. *Kidney Int.* (2024) 105:1239–53. 10.1016/j.kint.2024.01.040 38431216

[89] Tan J Dong L Jiang Z Tan L Luo X Pei G Probiotics ameliorate IgA nephropathy by improving gut dysbiosis and blunting NLRP3 signaling. *J Transl Med.* (2022) 20:382. 10.1186/s12967-022-03585-3 36038927 PMC9422169

[90] Ji J Jin W Liu S Jiao Z Li X. Probiotics, prebiotics, and postbiotics in health and disease. *MedComm.* (2023) 4:e420. 10.1002/mco2.420 37929014 PMC10625129

[91] Kim Y Mills D. Exploring the gut microbiome: Probiotics, prebiotics, synbiotics, and postbiotics as key players in human health and disease improvement. *Food Sci Biotechnol.* (2024) 33:2065–80. 10.1007/s10068-024-01620-1 39130661 PMC11315840

[92] Jiang Z Yang M Su W Mei L Li Y Guo Y Probiotics in piglet: From gut health to pathogen defense mechanisms. *Front Immunol.* (2024) 15:1468873. 10.3389/fimmu.2024.1468873 39559358 PMC11570287

[93] Chávez-Íñiguez J Ibarra-Estrada M Gallardo-González A Cisneros-Hernández A Claure-Del Granado R Chávez-Alonso G Probiotics in septic acute kidney injury, a double blind, randomized control trial. *Ren Fail.* (2023) 45:2260003. 10.1080/0886022X.2023.2260003 37724527 PMC10512773

[94] Ikeda Y Suga N Matsuda S. Efficacy of life protection probably from newly isolated bacteria against cisplatin-induced lethal toxicity. *Microorganisms.* (2023) 11:2246. 10.3390/microorganisms11092246 37764090 PMC10536890

[95] Miao H Liu F Wang Y Yu X Zhuang S Guo Y Targeting Lactobacillus johnsonii to reverse chronic kidney disease. *Signal Transduct Target Ther.* (2024) 9:195. 10.1038/s41392-024-01913-1 39098923 PMC11298530

[96] Ranganathan N Patel B Ranganathan P Marczely J Dheer R Chordia T Probiotic amelioration of azotemia in 5/6th nephrectomized Sprague-Dawley rats. *ScientificWorldJournal.* (2005) 5:652–60. 10.1100/tsw.2005.86 16127597 PMC5936581

[97] Zhou W Wu W Si Z Liu H Wang H Jiang H The gut microbe *Bacteroides* fragilis ameliorates renal fibrosis in mice. *Nat Commun.* (2022) 13:6081. 10.1038/s41467-022-33824-6 36241632 PMC9568537

[98] Stepanova N. Probiotic interventions in peritoneal dialysis: A review of underlying mechanisms and therapeutic potentials. *World J Nephrol.* (2024) 13:98719. 10.5527/wjn.v13.i4.98719 39723354 PMC11572655

[99] Rossi R Mainardi E. Prebiotics and probiotics supplementation in pigs as a model for human gut health and disease. *Biomolecules.* (2025) 15:665. 10.3390/biom15050665 40427557 PMC12109129

[100] Rauch C Mika A McCubbin A Huschtscha Z Costa R. Effect of prebiotics, probiotics, and synbiotics on gastrointestinal outcomes in healthy adults and active adults at rest and in response to exercise-A systematic literature review. *Front Nutr.* (2022) 9:1003620. 10.3389/fnut.2022.1003620 36570133 PMC9768503

[101] Gibson G Hutkins R Sanders M Prescott S Reimer R Salminen S Expert consensus document: The International scientific association for probiotics and prebiotics (ISAPP) consensus statement on the definition and scope of prebiotics. *Nat Rev Gastroenterol Hepatol.* (2017) 14:491–502. 10.1038/nrgastro.2017.75 28611480

[102] Esgalhado M Kemp J Azevedo R Paiva B Stockler-Pinto M Dolenga C Could resistant starch supplementation improve inflammatory and oxidative stress biomarkers and uremic toxins levels in hemodialysis patients? A pilot randomized controlled trial. *Food Funct.* (2018) 9:6508–16. 10.1039/c8fo01876f 30468238

[103] Sueyoshi M Fukunaga M Mei M Nakajima A Tanaka G Murase T Effects of lactulose on renal function and gut microbiota in adenine-induced chronic kidney disease rats. *Clin Exp Nephrol.* (2019) 23:908–19. 10.1007/s10157-019-01727-4 30895529 PMC6555783

[104] Singh V Yeoh B Chassaing B Xiao X Saha P Aguilera Olvera R Dysregulated microbial fermentation of soluble fiber induces cholestatic liver cancer. *Cell.* (2018) 175:679–94.e22. 10.1016/j.cell.2018.09.004 30340040 PMC6232850

[105] Nakabayashi I Nakamura M Kawakami K Ohta T Kato I Uchida K Effects of synbiotic treatment on serum level of p-cresol in haemodialysis patients: A preliminary study. *Nephrol Dial Transplant.* (2011) 26:1094–8. 10.1093/ndt/gfq624 20929916

[106] Rossi M Johnson D Morrison M Pascoe E Coombes J Forbes J Synbiotics easing renal failure by improving gut microbiology (SYNERGY): A randomized trial. *Clin J Am Soc Nephrol.* (2016) 11:223–31. 10.2215/CJN.05240515 26772193 PMC4741035

[107] Haghighat N Mohammadshahi M Shayanpour S Haghighizadeh M. Effects of synbiotics and probiotics supplementation on serum levels of endotoxin, heat shock protein 70 antibodies and inflammatory markers in hemodialysis patients: A randomized double-blinded controlled trial. *Probiotics Antimicrob Proteins.* (2020) 12:144–51. 10.1007/s12602-018-9509-5 30617950

[108] Ma L Li J Zhang X Zhang W Jiang C Yang B Chinese botanical drugs targeting mitophagy to alleviate diabetic kidney disease, a comprehensive review. *Front Pharmacol.* (2024) 15:1360179. 10.3389/fphar.2024.1360179 38803440 PMC11128677

[109] Zheng S Xu Y Zhang Y Long C Chen G Jin Z Efficacy and safety of traditional Chinese medicine decoction as an adjuvant treatment for diabetic nephropathy: A systematic review and meta-analysis of randomized controlled trials. *Front Pharmacol.* (2024) 15:1327030. 10.3389/fphar.2024.1327030 38783937 PMC11111926

[110] Long C Feng H Liu Z Li Z Liu J Jiang Y Efficacy of traditional Chinese medicine injection for diabetic kidney disease: A network meta analysis and systematic review. *Front Pharmacol.* (2023) 14:1028257. 10.3389/fphar.2023.1028257 36874023 PMC9981802

[111] Guo Z Wu X Zhang S Yang J Miao H Zhao Y. Poria cocos: Traditional uses, triterpenoid components and their renoprotective pharmacology. *Acta Pharmacol Sin.* (2024) 46:836–51. 10.1038/s41401-024-01404-7 39482471 PMC11950336

[112] Vagopoulou A Theofilis P Karasavvidou D Haddad N Makridis D Tzimikas S Pilot study on the effect of flavonoids on arterial stiffness and oxidative stress in chronic kidney disease. *World J Nephrol.* (2024) 13:95262. 10.5527/wjn.v13.i3.95262 39351188 PMC11439090

[113] Zhong X Jia J Tan R Wang L. Hederagenin improves adriamycin-induced nephropathy by inhibiting the JAK/STAT signaling pathway. *Integr Med Nephrol Androl.* (2024) 11:e22–00016. 10.1097/imna-d-22-00016

[114] Wang B Yang L Yang L Liang Y Guo F Fu P Fisetin ameliorates fibrotic kidney disease in mice via inhibiting ACSL4-mediated tubular ferroptosis. *Acta Pharmacol Sin.* (2024) 45:150–65. 10.1038/s41401-023-01156-w 37696989 PMC10770410

[115] Yang Y Wu C. Traditional Chinese medicine in ameliorating diabetic kidney disease via modulating gut microbiota. *Integr Med Nephrol Androl.* (2021) 8:8. 10.4103/imna.imna_28_21 39968893

[116] Li J Xu Y Sun T Zhang X Liang H Lin W Exploration of the pathogenesis of nephrotic syndrome and traditional Chinese medicine intervention based on gut microbiota. *Front Immunol.* (2024) 15:1430356. 10.3389/fimmu.2024.1430356 39717782 PMC11663840

[117] Zheng L Luo M Zhou H Chen J. Natural products from plants and microorganisms: Novel therapeutics for chronic kidney disease via gut microbiota regulation. *Front Pharmacol.* (2022) 13:1068613. 10.3389/fphar.2022.1068613 36733377 PMC9887141

[118] Zhang C Yue D Wang D Wu F. Effects of Bifidobacterium bifidum tetragonum tablets and Jin Gui Ren Qi Pill on intestinal flora and metabolism in patients with diabetic kidney disease. *Front Pharmacol.* (2024) 15:1346168. 10.3389/fphar.2024.1346168 39139646 PMC11319841

[119] Wang L Xu A Wang J Fan G Liu R Wei L The effect and mechanism of Fushen Granule on gut microbiome in the prevention and treatment of chronic renal failure. *Front Cell Infect Microbiol.* (2023) 13:1334213. 10.3389/fcimb.2023.1334213 38274729 PMC10808756

[120] Zhao H Zhao T Li P. Gut microbiota-derived metabolites: A new perspective of traditional Chinese medicine against diabetic kidney disease. *Integr Med Nephrol Androl.* (2024) 11:e23–00024. 10.1097/imna-d-23-00024

[121] Cai T Ye X Li R Chen H Wang Y Yong H Resveratrol modulates the gut microbiota and inflammation to protect against diabetic nephropathy in mice. *Front Pharmacol.* (2020) 11:1249. 10.3389/fphar.2020.01249 32973502 PMC7466761

[122] Zhu X Deng Z Cao Y Zhou Z Sun W Liu C Resveratrol prevents Drp1-mediated mitochondrial fission in the diabetic kidney through the PDE4D/PKA pathway. *Phytother Res.* (2023) 37:5916–31. 10.1002/ptr.8004 37767771

[123] Wang R Yuan W Li L Lu F Zhang L Gong H Resveratrol ameliorates muscle atrophy in chronic kidney disease via the axis of SIRT1/FoxO1. *Phytother Res.* (2022) 36:3265–75. 10.1002/ptr.7499 35606908

[124] Yan H Zhang Y Lin X Huang J Zhang F Chen C Resveratrol improves diabetic kidney disease by modulating the gut microbiota-short chain fatty acids axis in db/db mice. *Int J Food Sci Nutr.* (2024) 75:264–76. 10.1080/09637486.2024.2303041 38238900

[125] Alvarenga L Cardozo L Ribeiro-Alves M Damasceno N Berretta A Lima J Effects of turmeric extract supplementation on the lipid and lipoprotein subfraction profile in hemodialysis patients: A randomised, double-blind, crossover and controlled trial. *Phytother Res.* (2023) 37:3424–37. 10.1002/ptr.7814 37042623

[126] Shi H Chen L Wang C Zhao Y Wang Y Xue C Docosahexaenoic acid-acylated curcumin diester alleviates cisplatin-induced acute kidney injury by regulating the effect of gut microbiota on the lipopolysaccharide- and trimethylamine- N-oxide-mediated PI3K/Akt/NF-κB signaling pathway in mice. *Food Funct.* (2022) 13:6103–17. 10.1039/d1fo04178a 35575345

[127] Lyu X Zhang T Ye Z Chen C. Astragaloside IV mitigated diabetic nephropathy by restructuring intestinal microflora and ferroptosis. *Mol Nutr Food Res.* (2024) 68:e2300734. 10.1002/mnfr.202300734 38389170

[128] Zhong Z Zhang Y Wei Y Li X Ren L Li Y Fucoidan improves early stage diabetic nephropathy via the gut microbiota-mitochondria axis in high-fat diet-induced diabetic mice. *J Agric Food Chem.* (2024) 72:9755–67. 10.1021/acs.jafc.3c08503 38635872

[129] Zhang M Yang L Zhu M Yang B Yang Y Jia X Moutan cortex polysaccharide ameliorates diabetic kidney disease via modulating gut microbiota dynamically in rats. *Int J Biol Macromol.* (2022) 206:849–60. 10.1016/j.ijbiomac.2022.03.077 35307460

[130] Liu W Xu S Zhang B Sun X. Ramulus mori (Sangzhi) alkaloids alleviate diabetic nephropathy through improving gut microbiota disorder. *Nutrients.* (2024) 16:2346. 10.3390/nu16142346 39064789 PMC11280480

[131] Porcari S Benech N Valles-Colomer M Segata N Gasbarrini A Cammarota G Key determinants of success in fecal microbiota transplantation: From microbiome to clinic. *Cell Host Microbe.* (2023) 31:712–33. 10.1016/j.chom.2023.03.020 37167953

[132] Smits L Bouter K de Vos W Borody T Nieuwdorp M. Therapeutic potential of fecal microbiota transplantation. *Gastroenterology.* (2013) 145:946–53. 10.1053/j.gastro.2013.08.058 24018052

[133] Li Y Su X Gao Y Lv C Gao Z Liu Y The potential role of the gut microbiota in modulating renal function in experimental diabetic nephropathy murine models established in same environment. *Biochim Biophys Acta Mol Basis Dis.* (2020) 1866:165764. 10.1016/j.bbadis.2020.165764 32169506

[134] Li S Zhu A Benes V Costea P Hercog R Hildebrand F Durable coexistence of donor and recipient strains after fecal microbiota transplantation. *Science.* (2016) 352:586–9. 10.1126/science.aad8852 27126044

[135] Ray K. Gut microbiota: FMT - enduring strains. *Nat Rev Gastroenterol Hepatol.* (2016) 13:376. 10.1038/nrgastro.2016.84 27188820

[136] Bennet J Brinkman M. Treatment of ulcerative colitis by implantation of normal colonic flora. *Lancet.* (1989) 1:164. 10.1016/s0140-6736(89)91183-5 2563083

[137] Aas J Gessert C Bakken J. Recurrent Clostridium difficile colitis: Case series involving 18 patients treated with donor stool administered via a nasogastric tube. *Clin Infect Dis.* (2003) 36:580–5. 10.1086/367657 12594638

[138] van Nood E Vrieze A Nieuwdorp M Fuentes S Zoetendal E de Vos W Duodenal infusion of donor feces for recurrent Clostridium difficile. *N Engl J Med.* (2013) 368:407–15. 10.1056/NEJMoa1205037 23323867

[139] Surawicz C Brandt L Binion D Ananthakrishnan A Curry S Gilligan P Guidelines for diagnosis, treatment, and prevention of Clostridium difficile infections. *Am J Gastroenterol.* (2013) 108:478–98. 10.1038/ajg.2013.4 23439232

[140] Ruszkowski J Kachlik Z Walaszek M Storman D Podkowa K Garbarczuk P Fecal microbiota transplantation from patients into animals to establish human microbiota-associated animal models: A scoping review. *J Transl Med.* (2025) 23:662. 10.1186/s12967-025-06645-6 40528217 PMC12172294

[141] Wrzosek L Ciocan D Borentain P Spatz M Puchois V Hugot C Transplantation of human microbiota into conventional mice durably reshapes the gut microbiota. *Sci Rep.* (2018) 8:6854. 10.1038/s41598-018-25300-3 29717179 PMC5931539

[142] Li Y Cao W Gao N Zhao X Chen W. Consistent alterations of human fecal microbes after transplantation into germ-free mice. *Genomics Proteomics Bioinformatics.* (2022) 20:382–93. 10.1016/j.gpb.2020.06.024 34118462 PMC9684084

[143] Lee C Steiner T Petrof E Smieja M Roscoe D Nematallah A Frozen vs fresh fecal microbiota transplantation and clinical resolution of diarrhea in patients with recurrent clostridium difficile infection: A randomized clinical trial. *JAMA.* (2016) 315:142–9. 10.1001/jama.2015.18098 26757463

[144] Bassis C Moore N Lolans K Seekatz A Weinstein R Young V Comparison of stool versus rectal swab samples and storage conditions on bacterial community profiles. *BMC Microbiol.* (2017) 17:78. 10.1186/s12866-017-0983-9 28359329 PMC5374586

[145] Li X Shi X Yao Y Shen Y Wu X Cai T Effects of stool sample preservation methods on gut microbiota biodiversity: New original data and systematic review with meta-analysis. *Microbiol Spectr.* (2023) 11:e0429722. 10.1128/spectrum.04297-22 37093040 PMC10269478

[146] Zheng L Ji Y Wen X Duan S. Fecal microbiota transplantation in the metabolic diseases: Current status and perspectives. *World J Gastroenterol.* (2022) 28:2546–60. 10.3748/wjg.v28.i23.2546 35949351 PMC9254144

[147] Secombe K Al-Qadami G Subramaniam C Bowen J Scott J Van Sebille Y Guidelines for reporting on animal fecal transplantation (GRAFT) studies: Recommendations from a systematic review of murine transplantation protocols. *Gut Microbes.* (2021) 13:1979878. 10.1080/19490976.2021.1979878 34586011 PMC8489962

[148] Johnson J Spakowicz D Hong B Petersen L Demkowicz P Chen L Evaluation of 16S rRNA gene sequencing for species and strain-level microbiome analysis. *Nat Commun.* (2019) 10:5029. 10.1038/s41467-019-13036-1 31695033 PMC6834636

[149] Smillie C Sauk J Gevers D Friedman J Sung J Youngster I Strain tracking reveals the determinants of bacterial engraftment in the human gut following fecal microbiota transplantation. *Cell Host Microbe.* (2018) 23:229–40.e5. 10.1016/j.chom.2018.01.003 29447696 PMC8318347

[150] Jaworska K Koper M Ufnal M. Gut microbiota and renin-angiotensin system: A complex interplay at local and systemic levels. *Am J Physiol Gastrointest Liver Physiol.* (2021) 321:G355–66. 10.1152/ajpgi.00099.2021 34405730 PMC8486428

[151] Karbach S Schönfelder T Brandão I Wilms E Hörmann N Jäckel S Gut microbiota promote angiotensin II-Induced arterial hypertension and vascular dysfunction. *J Am Heart Assoc.* (2016) 5:e003698. 10.1161/JAHA.116.003698 27577581 PMC5079031

[152] Miao H Wang Y Su W Zou L Zhuang S Yu X Sirtuin 6 protects against podocyte injury by blocking the renin-angiotensin system by inhibiting the Wnt1/β-catenin pathway. *Acta Pharmacol Sin.* (2024) 45:137–49. 10.1038/s41401-023-01148-w 37640899 PMC10770168

[153] Xu K Guo Y Wang Y Ren Y Low V Cho S Decreased *Enterobacteriaceae* translocation due to gut microbiota remodeling mediates the alleviation of premature aging by a high-fat diet. *Aging Cell.* (2023) 22:e13760. 10.1111/acel.13760 36567449 PMC9924944

[154] Jiang S Shui Y Cui Y Tang C Wang X Qiu X Gut microbiota dependent trimethylamine N-oxide aggravates angiotensin II-induced hypertension. *Redox Biol.* (2021) 46:102115. 10.1016/j.redox.2021.102115 34474396 PMC8408632

[155] Caggiano G Stasi A Franzin R Fiorentino M Cimmarusti M Deleonardis A Fecal microbiota transplantation in reducing uremic toxins accumulation in kidney disease: Current understanding and future perspectives. *Toxins (Basel).* (2023) 15:115. 10.3390/toxins15020115 36828429 PMC9965504

[156] Bian J Liebert A Bicknell B Chen X Huang C Pollock C. Faecal microbiota transplantation and chronic kidney disease. *Nutrients.* (2022) 14:2528. 10.3390/nu14122528 35745257 PMC9228952

[157] Liu X Zhang M Wang X Liu P Wang L Li Y Fecal microbiota transplantation restores normal fecal composition and delays malignant development of mild chronic kidney disease in rats. *Front Microbiol.* (2022) 13:1037257. 10.3389/fmicb.2022.1037257 36532422 PMC9748282

[158] Burrello C Garavaglia F Cribiù F Ercoli G Lopez G Troisi J Therapeutic faecal microbiota transplantation controls intestinal inflammation through IL10 secretion by immune cells. *Nat Commun.* (2018) 9:5184. 10.1038/s41467-018-07359-8 30518790 PMC6281577

[159] Cammarota G Ianiro G Tilg H Rajiliæ-Stojanoviæ M Kump P Satokari R European consensus conference on faecal microbiota transplantation in clinical practice. *Gut.* (2017) 66:569–80. 10.1136/gutjnl-2016-313017 28087657 PMC5529972

[160] Lauriero G Abbad L Vacca M Celano G Chemouny J Calasso M Fecal microbiota transplantation modulates renal phenotype in the humanized mouse model of IgA nephropathy. *Front Immunol.* (2021) 12:694787. 10.3389/fimmu.2021.694787 34712223 PMC8546224

[161] Bastos R Simplício-Filho A Sávio-Silva C Oliveira L Cruz G Sousa E Fecal microbiota transplant in a Pre-clinical model of Type 2 diabetes mellitus, obesity and diabetic kidney disease. *Int J Mol Sci.* (2022) 23:3742. 10.3390/ijms23073842 35409202 PMC8998923

[162] Knauf F Brewer J Flavell R. Immunity, microbiota and kidney disease. *Nat Rev Nephrol.* (2019) 15:263–74. 10.1038/s41581-019-0118-7 30796361

[163] Jung C Yoo T. Pathophysiologic mechanisms and potential biomarkers in diabetic kidney disease. *Diabetes Metab J.* (2022) 46:181–97. 10.4093/dmj.2021.0329 35385633 PMC8987689

[164] Lau W Chang Y Vaziri N. The consequences of altered microbiota in immune-related chronic kidney disease. *Nephrol Dial Transplant.* (2021) 36:1791–8. 10.1093/ndt/gfaa087 32437554 PMC8633451

[165] Luo L Luo J Cai Y Fu M Li W Shi L Inulin-type fructans change the gut microbiota and prevent the development of diabetic nephropathy. *Pharmacol Res.* (2022) 183:106367. 10.1016/j.phrs.2022.106367 35882293

[166] Lu J Chen P Zhang J Li X Wang G Yuan B GPR43 deficiency protects against podocyte insulin resistance in diabetic nephropathy through the restoration of AMPKα activity. *Theranostics.* (2021) 11:4728–42. 10.7150/thno.56598 33754024 PMC7978296

[167] Shang J Cui W Guo R Zhang Y Wang P Yu W The harmful intestinal microbial community accumulates during DKD exacerbation and microbiome-metabolome combined validation in a mouse model. *Front Endocrinol (Lausanne).* (2022) 13:964389. 10.3389/fendo.2022.964389 36601003 PMC9806430

[168] Yuan X Qing J Zhi W Wu F Yan Y Li Y. Gut and respiratory microbiota landscapes in IgA nephropathy: A cross-sectional study. *Ren Fail.* (2024) 46:2399749. 10.1080/0886022X.2024.2399749 39248406 PMC11385635

[169] De Angelis M Montemurno E Piccolo M Vannini L Lauriero G Maranzano V Microbiota and metabolome associated with immunoglobulin A nephropathy (IgAN). *PLoS One.* (2014) 9:e99006. 10.1371/journal.pone.0099006 24922509 PMC4055632

[170] Zhao J Bai M Yang X Wang Y Li R Sun S. Alleviation of refractory IgA nephropathy by intensive fecal microbiota transplantation: The first case reports. *Ren Fail.* (2021) 43:928–33. 10.1080/0886022X.2021.1936038 34134605 PMC8901287

[171] Zhi W Song W Abdi Saed Y Wang Y Li Y. Fecal capsule as a therapeutic strategy in IgA Nephropathy: A brief report. *Front Med (Lausanne).* (2022) 9:914250. 10.3389/fmed.2022.914250 35647000 PMC9133370

[172] Zhi W Li A Wang Q Yuan X Qing J Zhang C Safety and efficacy assessment of fecal microbiota transplantation as an adjunctive treatment for IgA nephropathy: An exploratory clinical trial. *Sci Rep.* (2024) 14:22935. 10.1038/s41598-024-74171-4 39358432 PMC11446926

[173] Zhu Y He H Sun W Wu J Xiao Y Peng Y IgA nephropathy: Gut microbiome regulates the production of hypoglycosilated IgA1 via the TLR4 signaling pathway. *Nephrol Dial Transplant.* (2024) 39:1624–41. 10.1093/ndt/gfae052 38402460 PMC11427068

[174] Wang M Yang J Fang X Lin W Yang Y. Membranous nephropathy: Pathogenesis and treatments. *MedComm.* (2020) 2024:e614. 10.1002/mco2.614 38948114 PMC11214595

[175] Shi X Li Z Lin W Shi W Hu R Chen G Altered intestinal microbial flora and metabolism in patients with idiopathic membranous nephropathy. *Am J Nephrol.* (2023) 54:451–70. 10.1159/000533537 37793354

[176] Miao H Wang Y Yu X Zou L Guo Y Su W Lactobacillus species ameliorate membranous nephropathy through inhibiting the aryl hydrocarbon receptor pathway via tryptophan-produced indole metabolites. *Br J Pharmacol.* (2024) 181:162–79. 10.1111/bph.16219 37594378

[177] Shang J Zhang Y Guo R Liu W Zhang J Yan G Gut microbiome analysis can be used as a noninvasive diagnostic tool and plays an essential role in the onset of membranous nephropathy. *Adv Sci (Weinh).* (2022) 9:e2201581. 10.1002/advs.202201581 35975460 PMC9534961

[178] Zhou G Zeng J Peng L Wang L Zheng W Di W Fecal microbiota transplantation for membranous nephropathy. *CEN Case Rep.* (2021) 10:261–4. 10.1007/s13730-020-00560-z 33387212 PMC8019434

[179] Zhi W Yuan X Song W Jin G Li Y. Fecal microbiota transplantation may represent a good approach for patients with focal segmental glomerulosclerosis: A brief report. *J Clin Med.* (2022) 11:6700. 10.3390/jcm11226700 36431177 PMC9697655

